# Myiasis by *Cordylobia anthropophaga* and *C. rodhaini* (Diptera: Calliphoridae) in Polish travelers to Africa with new molecular data

**DOI:** 10.1093/jme/tjaf006

**Published:** 2025-01-25

**Authors:** Beata Biernat, Paweł Gładysz, Anna Kuna, Małgorzata Sulima, Martyna Bykowska-Tumasz, Elżbieta Sontag

**Affiliations:** Division of Tropical Parasitology, Institute of Maritime and Tropical Medicine, Medical University of Gdańsk, Powstania Styczniowego 9B, Gdynia 81-519, Poland; Division of Tropical Parasitology, Institute of Maritime and Tropical Medicine, Medical University of Gdańsk, Powstania Styczniowego 9B, Gdynia 81-519, Poland; Department of Tropical and Parasitic Diseases, University Centre for Maritime and Tropical Medicine, Medical University of Gdańsk, Powstania Styczniowego 9B, Gdynia 81-519, Poland; Department of Tropical and Parasitic Diseases, University Centre for Maritime and Tropical Medicine, Medical University of Gdańsk, Powstania Styczniowego 9B, Gdynia 81-519, Poland; Department of Tropical and Parasitic Diseases, University Centre for Maritime and Tropical Medicine, Medical University of Gdańsk, Powstania Styczniowego 9B, Gdynia 81-519, Poland; Department of Invertebrate Zoology and Parasitology, Faculty of Biology, University of Gdańsk, Wita Stwosza 59, Gdańsk 80-308, Poland

**Keywords:** travel medicine, myiasis, tumbu fly, Lund’s fly

## Abstract

Myiasis is a parasitic infestation of soft vertebrate tissues by larval stages of Diptera. We briefly described the lesion-causing genus *Cordylobia* Grünberg (Diptera: Calliphoridae). Three Polish travelers to Uganda, Gambia, and Senegal returned with furuncular myiasis. To identify the third-instar larvae removed from their skin, we examined the morphological features of the 3 specimens and sequenced a 5’ barcoding fragment of the cytochrome c oxidase subunit I gene (COI-5P). One larva was identified as *C. rodhaini* Gedoelst, and 2 larvae were identified as *C. anthropophaga* (Blanchard). We were the first to submit the COI-5P of *C. rodhaini* to GenBank and the Barcode of Life Database. This is the first record of the importation of *C. anthropophaga* and the second record of the importation of *C. rodhaini* to Poland.

## Introduction

There are several species of dipteran flies whose larvae can parasitize in human skin and cavities. The most common type of the resulting disease condition is cutaneous myiasis manifesting as a wound or traumatic myiasis (larvae feed on living and necrotic tissue), creeping myiasis (larvae migrate under the skin), or furuncular myiasis (larvae remain in the skin and produce boil-like nodules) (Hall and Smith 1993).

Among African Calliphoridae, the genus *Cordylobia* Grünberg, 1903 (Diptera: Calliphoridae) comprises 4 species: *Cordylobia anthropophaga* (Blanchard, 1872) (tumbu fly), *Cordylobia rodhaini* Gedoelst, 1910 (Lund’s fly), *Cordylobia ruandae* Fain, 1953 (forest mouse fly), and *Cordylobia roubaudi* Villeneuve, 1929 (Rognes 2011). Their larvae are obligatory parasites of wild and domestic mammals and cause furuncular myiasis. *Cordylobia ruandae* is only known to parasitize the forest mouse *Grammomys dolichurus surdaster* (Thomas & Wroughton, 1908) (Rodentia: Muridae) (Zumpt 1965). When it comes to *C. roubaudi*, the species’ biology remains unknown, but adults are generally found in the entrances of warthog burrows (Zumpt 1965).

*Cordylobia anthropophaga* occurs in East, Central, and West Africa (Francesconi and Lupi 2012) and Saudi Arabia (Afifi et al. 2015), though, some tourists acquired the parasite in southern Spain (Laurence and Herman 1973) and Portugal (Curtis et al. 2006). Curiously, there were Londoners infected with *C. anthropophaga* most likely through contact with the contaminated clothes of relatives who had recently visited Africa (Baily and Moody 1985, Whitehorn et al. 2010).

The Tumbu fly prefers a warm and humid environment. Adult females lay eggs on dry, shaded sand (often contaminated with urine and feces), on laundry hung out to dry out of direct sunlight, or on dirty clothes (Zumpt 1965, Hall and Wall 1995). After hatching, larvae penetrate the host skin, producing itchy sores that develop into painful, boil-like lesions (intense inflammatory reaction), and proceed through 3 stages of larval development before entering the prepupal stage (Zumpt 1965, Francesconi and Lupi 2012). The post-feeding third-instar larva leaves the host and drops to the ground to develop into a mature fly (Hall and Smith 1993). Like other Calliphoridae, adult flies feed on plant juice and animal feces (Zumpt 1965). They are most active in the morning and late afternoon and rest in dark places during the day (Zumpt 1965).

*Cordylobia rodhaini* resembles the tumbu fly in appearance and life cycle but infests humans much less frequently. It occurs in tropical Africa, especially in areas covered by the rainforest (Zumpt 1965, Geary et al. 1999). Antelopes and the African giant rat *Cricetomys gambianus* Waterhouse, 1840 (Rodentia: Nesomyidae) are the main reservoirs of *C. rodhaini*, but the species targets a variety of mammals, including monkeys (Zumpt 1965). Human furuncular myiasis caused by *C. rodhaini* has been diagnosed in the east and west of Central Africa and Zimbabwe (Pezzi et al. 2015). The life history of *C. rodhaini* is similar to that of *C. anthropophaga*, except lesions caused by the immature stages can be bigger and more painful due to the larger size achieved by the larvae (Zumpt 1965, Francesconi and Lupi 2012).

This study aimed to use a mitochondrial marker, cytochrome c oxidase subunit I (COI), to confirm a morphology-based identification of 3 myiasis-causing larvae of blow flies extracted from the skin of Polish travelers to Africa.

## Materials and Methods

Three travelers returned to Poland from their respective trips to Uganda (July–August 2018), Gambia (March 2019), and Gambia and Senegal (January 2020), with single skin lesions typical of furuncular myiasis, each containing 1 larva. They did not visit other African countries during their journeys.

### Morphological Identification

The 3 larvae—UG-01 (Uganda), GM-01 (Gambia), and GMSN-01 (Gambia/Senegal)—were preserved in ethanol 70% (Chempur, cat. no. 113964201, Piekary Śląskie, Poland). We examined the specimens with a Leica M205A stereomicroscope and photographed them with a Leica DM6000 digital camera operated with Leica Application Suite 3.7 (Leica Microsystems, Wetzlar, Germany). Posterior parts of specimens UG-01 and GMSN-01 were macerated in potassium hydroxide 10% (Chempur, cat. no. 527468006, Piekary Śląskie, Poland) at room temperature for 15 min and thoroughly rinsed with ethanol 70%. Softened muscles were removed with fine forceps and a needle. The analysis at the species level was based on 2 taxonomic keys (Zumpt 1965, Hall and Smith 1993).

### Molecular Identification

Total genomic DNA was extracted from segments VI–VIII with a commercially available increased-efficiency kit for DNA purification from tissue and cell culture, Genomic Mini AX Tissue (A&A Biotechnology, cat. no. 056-60, Gdańsk, Poland). The amplification of a 648-bp 5’ fragment of cytochrome c oxidase subunit I mitochondrial gene (COI-5P) was performed in a total reaction volume of 25 µl using 12.5 µl of PCR Mix Plus HGC (A&A Biotechnology, cat. no. 2005-100G, Gdańsk, Poland), 0.4 µM of forward primer cocktail C_LepFolF, 0.4 µM of reverse primer cocktail C_LepFolR (Hernández-Triana et al. 2014) (Thermo Fisher Scientific Inc., UK), and 14–104 ng of the extracted DNA. The polymerase chain reaction (PCR) conditions were as follows: 95 °C for 4 min followed by 35 cycles of 95 °C for 30 s, 46 °C for 30 s, and 72 °C for 1 min, with the final extension at 72 °C for 10 min.

Bidirectional Sanger sequencing of size-verified PCR products was outsourced to Macrogen Europe (Amsterdam). The results were analyzed in Geneious Prime 2025.0.2 (https://www.geneious.com/, GraphPad Software, LLC (d.b.a. Geneious), Boston, MA, USA). Raw sequences were automatically trimmed (error probability limit of 0.01). Consensus sequences from forward and reverse reads were assembled and processed per the Data Standards for Barcode Records in the International Nucleotide Sequence Database Collaboration (Hanner 2012). Quality-checked COI-5P sequences were scanned for open reading frames to detect internal stop codons typical of nuclear copies of mitochondrial genes and compared with GenBank deposits using the Basic Local Alignment Search Tool (BLAST) (https://blast.ncbi.nlm.nih.gov/Blast.cgi). They are available in GenBank (UG-01: OP348988, GM-01: OP345031, GMSN-01: OP345032) and the Barcode of Life Data System (UG-01: PGCOR002-22, GM-01: PGCOR001-22).

## Results

### Morphological Identification

**Fig. 1.**
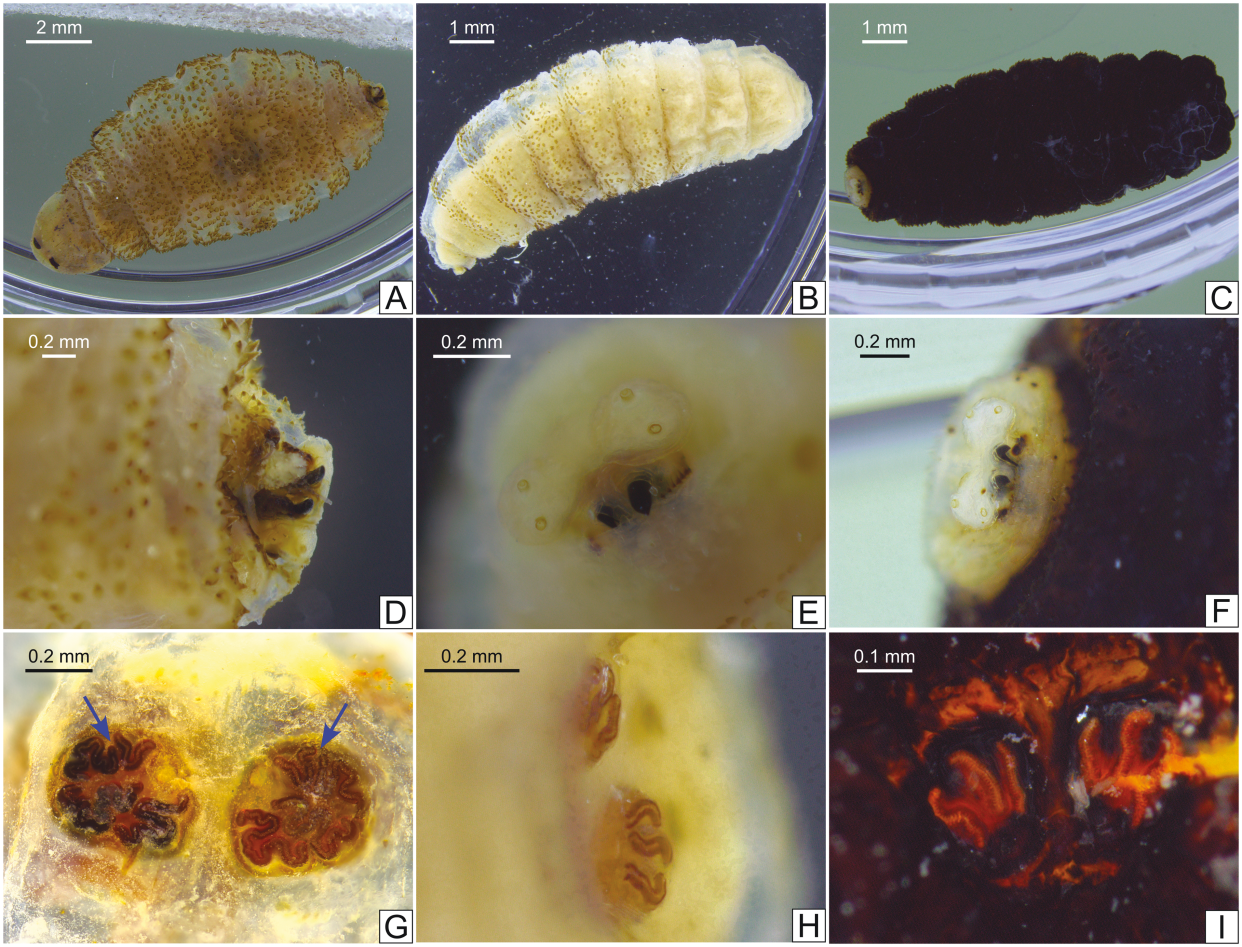
Morphological identification of third-instar larvae of *Cordylobia rodhaini* (sample UG-01) and *Cordylobia anthropophaga* (sample GM-01 and sample GMSN-01). A) Larva of *C. rodhaini* in toto, sample UG-01. B) Larva of *C. anthropophaga* in toto, sample GM-01. C) Larva of *C. anthropophaga* in toto, sample GMSN-01. D) Anterior part of the larva in A. E) Anterior part of the larva in B. F) Anterior part of the larva in C. G) Posterior part of the larva in A, showing the posterior spiracles (arrows). H) Posterior part of the larva in B, showing the posterior spiracles. I) Posterior part of the larva in C, showing the posterior spiracles.

The larvae were identified as belonging to the genus *Cordylobia* because the specimens presented cuticle covered with spines (Fig. 1A–C), 2 black mouth hooks (Fig. 1D–F), and posterior spiracles were not widely separated and had serpentine slits. Posterior peritremes had 3 sinuous or tortuous slits (Fig. 1G–I).

Species analysis was achieved by assessing the shape of posterior peritreme slits. In the case of *C. rodhaini*, the posterior peritreme slits are long and very tortuous, and at least 1 shows fragmentation in 2 (Fig. 1G). In contrast, in *C. anthropophaga*, the posterior peritreme slits are sinuous (Fig. 1H and I).

### Molecular Identification

We successfully amplified and sequenced the COI-5P fragment for all 3 specimens. The percentage of untrimmed bases of Q ≥ 20 in consensus sequences (663 bp) was as follows: UG-01, 98.0%; GM-01, 98.8%; GMSN-01, 99.5%. UG-01 matched a COI-5P record of an unidentified *Cordylobia* species in 98.08% (OQ024673.1, 588 bp, query cover: 86.00%) and a Cameroonian *C. anthropophaga* in 93.67% (FR719158.1, 1,562 bp, query cover: 100%). GM-01 and GMSN-01 exhibited 99.70% similarity to a COI-5P sequence of *C. anthropophaga* (FR719158.1, 1,562 bp, query cover: 100%). The expect value (number of expected random hits) of 100 sequences producing significant alignments in each of the 3 BLAST searches was zero.

## Discussion

Based on the literature review by Jallow et al. (2024), parasitism by *Cordylobia* spp. in humans is underreported. *Cordylobia anthropophaga* is the most common cause of myiasis in sub-Saharan Africa (104 out of 157 cases published from 1959 to 2022). In the same period, there were 16 reports of myiasis due to *C. rodhaini* infection. According to a different literature review, between 1902 and 2015, 25 cases of human furuncular myiasis caused by *C. rodhaini* were reported worldwide, all travelers to sub-Saharan Africa (Pezzi et al. 2015).

Only one previous imported case of *C. rodhaini* skin invasion was reported from Poland (Waśniowski and Rehlis 2006), and no case of imported myiasis caused by *C. anthropophaga* has been reported to date from this country. Local tropical medicine doctors informed us that some patients remove larvae themselves without seeking medical attention. Thus, in our opinion, the incidence of importation into Poland is higher than indicated by the literature but goes unreported. The same may occur in other European countries. With the number of visitors to the tropics on the rise, exotic parasitic diseases are becoming an increasingly relevant issue for tourists from the temperate zone.

The wounds of all 3 travelers were clean, with no healing issues reported post-removal. The maturation process can cause discomfort and distress as the larva grows and wiggles in the wound. There is also a risk of bacterial infection. An occlusive dressing with petroleum jelly forces the insect to emerge from the skin due to asphyxia. The exposed parasite may be removed by vacuum extraction or squeezing (Boggild et al. 2002). In the *Cordylobia*-endemic regions, it is best to protect the skin against infestation with well-ironed clothes dried in direct sunlight.

The best match for UG-01 represented a larva from Cote d’Ivoire, removed from human skin. On the one hand, its resemblance to our sequence may stem from the intraspecific variability of *C. rodhaini*. On the other hand, it may belong to one of the sister species, *C. ruandae* or *C. roubaudi*. Neither the case nor the morphological features of the larva were reported. We inspected our specimen morphologically, documented *C. rodhaini* features, and obtained its valid barcode. Before this study, identifying *C. rodhaini* with a molecular marker was impossible due to the lack of reference. The acquired COI-5P sequence will be useful for investigating larval stages, especially damaged individuals missing species-specific features.

## Data Availability

Partial coding sequences of the cytochrome c oxidase subunit I gene obtained in this study are available in GenBank (UG-01: OP348988, GM-01: OP345031, GMSN-01: OP345032) and in the Barcode of Life Data System (UG-01: PGCOR002-22, GM-01: PGCOR001-22).

## CRediT Statement

B. Biernat: Conceptualization, Methodology, Investigation, Writing—original draft, Writing—review & editing, Supervision, Project Administration. P. Gładysz: Investigation, Methodology, Data curation, Writing—original draft, Writing—review & editing. A. Kuna: Conceptualization, Writing—review & editing. M. Sulima: Resources, Writing—review & editing. M. Bykowska-Tumasz: Resources, Writing—review & editing. E. Sontag: Investigation, Writing—review & editing.
